# Supplementary material for: Supervised Exercise in Water: Is It a Viable Alternative in Overweight/Obese People with or without Type 2 Diabetes? A Pilot Study
Source: Nutrients. 2022 Nov 23;14(23):4963. doi: 10.3390/nu14234963 (PMC9737856; doi:10.3390/nu14234963)
Supplement: Supplementary file 1 [file nutrients-14-04963-s001.zip › nutrients-2056957-SI.pdf]

Table S1. Anthropometric, blood pressure, and blood chemistry parameters values at baseline (T0) in all the sample (n = 38), overweight/obese with diabetes (n = 20), and over-weight/obese without diabetes (n = 18) subjects. Results are presented as mean and Standard Deviation (SD). Statistical significance was set for p values ≤.05.

Variables	All		overweight/obese with diabetes		overweight/obese without diabetes		T test
	Mean	SD	Mean	SD	Mean	SD	p
Weight (kg)	65.96	47.62	72.59	46.99	58.87	48.08	0.268
BMI (kg/m^2)	36.77	6.21	37.44	7.25	36.03	4.89	0.494
WC (cm)	115.82	12.89	118.85	15.62	112.44	8.11	0.128
SBP (mmhg)	136.08	11.85	136.75	13.11	135.29	10.53	0.715
DBP (mmhg)	81.89	7.67	82.50	7.86	81.18	7.61	0.608
Fasting blood glucose (mg/dL)	108.95	30.37	122.10	36.09	94.33	11.02	0.003
HbA1c (%)	6.36	1.22	6.91	1.46	5.78	0.48	0.004
Total cholesterol (mg/dL)	211.00	51.41	197.70	57.34	225.78	40.48	0.093
HDL (mg/dL)	49.45	9.79	49.25	10.65	49.67	9.04	0.898
LDL cholesterol (mg/dL)	132.41	42.30	114.72	43.62	152.07	31.48	0.005
Triglycerides (mg/dL)	152.68	82.58	169.65	92.95	133.83	66.88	0.186
Uric acid (mg/dL)	5.64	1.36	5.71	1.44	5.59	1.33	0.810

Legend: BMI= Body mass index; WC = waist circumference; SBP = Systolic blood pressure; DBP = Diastolic blood pressure; HbA1c= glycosylated haemoglobin; Hdl= High Density Lipoprotein; LDL= Low Density Lipoprotein.
